# Regulation of the germinal center response by nuclear receptors and implications for autoimmune diseases

**DOI:** 10.1111/febs.15312

**Published:** 2020-04-19

**Authors:** William J. Olson, Bojana Jakic, Natascha Hermann‐Kleiter

**Affiliations:** ^1^ Translational Cell Genetics Department of Pharmacology and Genetics Medical University of Innsbruck Austria; ^2^ Department of Immunology, Genetics and Pathology Uppsala University Sweden

**Keywords:** autoimmune disease, follicular T helper cells, germinal center B cells, germinal center response, nuclear receptor, SLE

## Abstract

The immune system plays an essential role in protecting the host from infectious diseases and cancer. Notably, B and T lymphocytes from the adaptive arm of the immune system can co‐operate to form long‐lived antibody responses and are therefore the main target in vaccination approaches. Nevertheless, protective immune responses must be tightly regulated to avoid hyper‐responsiveness and responses against self that can result in autoimmunity. Nuclear receptors (NRs) are perfectly adapted to rapidly alter transcriptional cellular responses to altered environmental settings. Their functional role is associated with both immune deficiencies and autoimmunity. Despite extensive linking of nuclear receptor function with specific CD4 T helper subsets, research on the functional roles and mechanisms of specific NRs in CD4 follicular T helper cells (Tfh) and germinal center (GC) B cells during the germinal center reaction is just emerging. We review recent advances in our understanding of NR regulation in specific cell types of the GC response and discuss their implications for autoimmune diseases such as systemic lupus erythematosus (SLE).

## Graphical abstract

Autoreactive germinal centers (GCs) are central to autoimmune diseases such as systemic lupus erythematosus (SLE). While the induction of SLE and autoreactive GCs is a multifactorial process, nuclear receptors are known to play an essential role in SLE; some are currently targets for SLE treatment, while others are potential new targets. In this review, we summarize the current literature on NRs in the context of germinal centers and SLE.

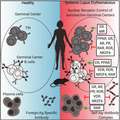

**Abbreviations**
BCR: B‐cell receptor
ER: estrogen receptor
GC: germinal center
GR: glucocorticoid receptor
ICOS: inducible T‐cell co‐stimulator
IL: interleukin
NR2F6: nuclear receptor subfamily 2 group F member 6
NRs: nuclear receptors
PPARs: peroxisome proliferator‐activated receptors
PR: progesterone receptor
RAR: retinoic acid receptor
ROR: retinoic acid receptor‐related orphan receptor
RXRs: retinoid X receptors
SLE: systemic lupus erythematosus
TCR: T‐cell receptor
Tfh: follicular T helper cell
Tfrs: follicular regulatory T cells
TR: testosterone receptor
Treg: regulatory T cells
VDR: vitamin D receptor

## Introduction

The immune system plays an essential role in protecting the host from infectious diseases caused by bacterial, viral, parasitic, or fungal pathogens but also during cancer immunosurveillance. Memory T and B cells from the adaptive arm of the immune system can successfully and quickly combat re‐infection or cancer outgrowth. However, protective immune responses must be restrained in order to avoid hyper‐responsiveness and responses against self that may result in autoimmunity due to the inefficiency of central tolerance or random mutations within germinal centers.

Both T and B lymphocytes collaborate in the production of high‐affinity antibodies through a process known as the germinal center (GC) reaction. The GC, in many ways, can be considered evolution in miniature [1]. Within the GC, B cells actively and randomly mutate B‐cell receptors (BCRs), a process termed somatic hyper‐mutation (SHM). B cells that acquire a mutation or mutations that confer increased affinity for antigen can gather more antigens through the BCR than lower affinity cells and, in turn, display more antigens on surface major histocompatibility complex (MHC). These cells are then preferentially selected by a limited number of CD4 follicular T helper (Tfh) cells through MHC:T‐cell receptor (TCR) interaction, coreceptor engagement, and cytokine signaling. Engagement with a Tfh cell permits the survival, proliferation, and further rounds of BCR mutation by the B cell. Alternatively, Tfh contact can result in B‐cell differentiation into antibody‐secreting plasma cells [2, 3]. Specialized CD4 T cells may suppress the GC response, as is the case for CD4 T regulatory (Treg) and follicular regulatory T (Tfr) cells [4]. These regulatory subsets are essential as both the generation and function of Tfh cells must be controlled to prevent the survival or plasma cell differentiation of self‐reactive B cells generated due to the random nature of SHM. Failure to adequately control GCs is the primary driver of several autoimmune diseases, including systemic lupus erythematosus (SLE) and rheumatoid arthritis [5, 6, 7].

Autoimmune diseases collectively afflict up to 10% of the developed world's population with a higher prevalence in women. The incidence of many autoimmune diseases, including systemic lupus erythematosus (SLE) and multiple sclerosis (MS), has been increasing over the past decade and is estimated to increase further in the coming decade(s) [8, 9, 10]. Despite advances in treating autoimmune diseases, treatment options often involve general immunosuppression, which can lead to adverse side effects. A deeper understanding of factors that influence the immunological causes of autoimmune diseases is needed in order to develop therapies that specifically target the pathogenic cell subsets and genes responsible for autoimmunity.

The nuclear receptor (NR) superfamily is one of the primary classes of therapeutic drug targets for human diseases [11]. Nuclear receptors, such as the glucocorticoid receptor (GR), the retinoid x receptors (RXR), the peroxisomal proliferator‐activated receptor α (PPARα), and gamma (PPARγ), are targets of already approved drugs for the treatment of autoimmunity, cancer, hyperlipidemia, or type 2 diabetes [12].

Within the T‐cell compartment, NRs regulate diverse mechanisms, including T‐cell receptor sensitivity, activation thresholds, surface receptor expression, cytokine expression, cell‐fate potential, metabolism, and migratory behavior. The mode of action of this receptor family is equally diverse but generally involves the binding of DNA hormone response elements (HREs) and recruitment of corepressors such as nuclear corepressor (NCoR) or silencing mediator of retinoic and thyroid receptors (SMRTs). Alternatively, NRs may recruit co‐activators, such as members of the steroid receptor co‐activator family (SRC) [13]. The exact function of a particular NR depends on a complex interplay of factors including the cell type, expression of other NRs, presence of ligands for the receptor, and cell conditions such as inflammation leading to changes in coreceptor or repressor recruitment and post‐translational modification of the receptors themselves [14].

Understanding the complex interaction between Tfh, Tfr cells, and GC B‐cell responses, the intensity and quality of an immune response and the specific role of NRs might improve both therapeutic options in autoimmune diseases as well as future vaccination strategies.

## Nuclear receptors

Nuclear receptors belong to a superfamily of structurally related ligand‐dependent transcription factors (TF)s consisting of 48 members in humans [15, 16]. NRs regulate a myriad of developmental and homeostatic processes in the steady state such as metabolism, circadian rhythm, immune cell homeostasis, reproduction, and pathological processes such as cancer, and metabolic, cardiovascular, and autoimmune diseases [12, 13, 17]. In parallel to their function as transcription factors, NRs have nongenomic functions, for instance, the activation of different signaling pathways such as the cAMP, Ca^2+^, or the MAPK cascade [12, 18, 19]. Based on ligand and DNA‐binding properties, the NR family has been divided into subfamilies, such as the classical steroid hormone receptor family which includes the glucocorticoid and the estrogen receptors, the orphan nuclear receptors for which no physiological ligand has yet been identified, as well as the adopted orphan nuclear receptors whose naturally occurring ligands were identified after the receptor had been characterized [11, 13, 20]. A more complex classification system for NRs was introduced by Germain et al. in which NRs are divided into seven subfamilies [21, 22].

The well‐conserved domain structure of NRs consists of an activation domain 1 (AF1), the central DNA‐binding domain (DBD), the hinge region, the ligand‐binding domain (LBD), and the activation function 2 (AF2) region [22, 23]. NR activity is mainly regulated via the conformational states of the AF‐2 region, which, in an agonist ligand‐dependent way, alters the ability of the LBD to recruit co‐activator proteins, whereas antagonistic ligands promote an inhibitory conformation of the LBD [24, 25].

**Fig. 1:**
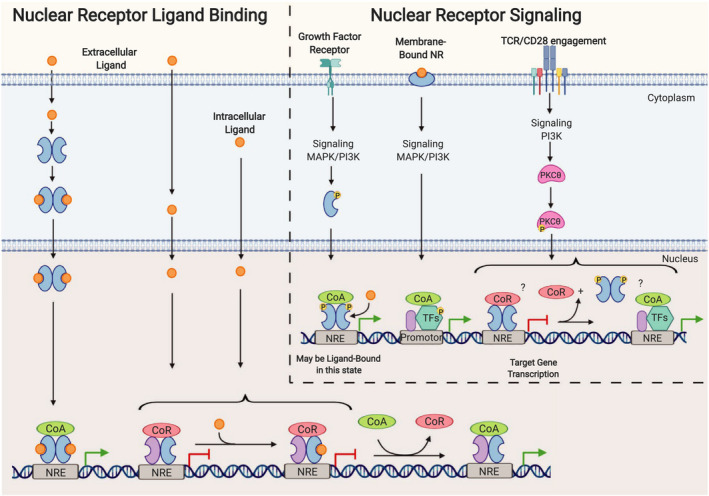
NR function and DNA binding can be affected by both ligand binding and surface receptor signaling. Nuclear receptors can be activated not only by extra‐ or intracellular ligands but also by surface receptor signaling via PI3K and MAPK. Ligands may bind NRs in the cytoplasm leading to translocation of NR‐ligand dimers into the nucleus resulting in the interaction of the NR‐ligand dimers with DNA nuclear response elements (NREs), recruitment of co‐activators (CoA), and subsequently gene transcription. Other NRs are constitutively bound to NREs and corepressors (CoR), and the ligand may diffuse into the nucleus resulting in reduced affinity for CoR and recruitment of CoAs followed by activation of gene transcription. PI3K and MAPK signal transduction can lead to NR phosphorylation and translocation into the nucleus, followed by binding to NREs and CoA recruitment and gene activation (i.e., ERα). Some NRs may be membrane‐associated, will bind ligand at this location, and activate PI3K and MAPK signaling, leading to target gene transcription through phosphorylation and activation of other TFs (i.e., GR). TCR and CD28 coreceptor engagement activates PKCθ through PI3K activation. PKCθ can translocate to the nucleus where it phosphorylates DNA‐bound NRs, resulting in the release of DNA. This presumably leaves the site open for other TF complex binding, resulting in target gene expression (i.e., NR2F6). Although the exact molecular mechanisms are not entirely clear for some NRs. Abbreviations: transcription factor (TF). Created with BioRender.

The binding of a ligand to the LBD induces a conformational change that results, in the case of the classical steroid hormones, in the translocation of ligand‐bound receptors into the nucleus followed by binding to nuclear receptor response elements in the DNA, subsequently altering gene expression [11, 12]. In contrast, other nuclear receptors, such as the peroxisome proliferator‐activated receptors (PPARs), are localized in the nucleus independent of ligand binding and constitutively interact with DNA response elements [11]. Multiple mechanisms of target gene control by NRs have been identified including recruitment of corepressors or co‐activators, as well as a mechanism known as tethering, that prevents recruitment of specific co‐activators (Fig. 1) [26]. The specificity of transcriptional activation by a given NR is achieved by epigenetic regulation of the genomic region as well as the tissue‐selective expression and recruitment of corepressors or co‐activators and post‐transcriptional modifications of both [18, 27]. The transcriptional activity of members of the NR family is associated with both immune deficiencies and autoimmunity and can exert incredibly diverse effects on cells of the innate and adaptive immune system [13, 26, 28]; surprisingly, detailed functional analysis of NRs in germinal center immunity is still lacking.

Nuclear receptors are known to play a significant role within the different CD4 helper subsets, for example, NRs are critical for control of the reciprocal differentiation potential of naive CD4 T cells into either pro‐inflammatory Th17 or tolerance‐inducing regulatory T cells. This aspect has been investigated in detail in the last decade, has been extensively reviewed recently, and will therefore not be covered in this review [29, 30, 31, 32, 33]. During pathogen encounter, vaccination responses, or autoimmune responses, the specific roles of NRs have been implicated in the formation of GCs and especially in the function of Tfh cells; however, detailed functional analysis is only now emerging.

## The germinal center reaction

Germinal centers (GCs) are micro‐anatomical structures that are formed within secondary lymphoid organs in response to T‐cell‐dependent antigen [34, 35]. They are critical for the production of high‐affinity antibodies and the long‐lived plasma cells that produce them; additionally, memory B and T cells are a product of this reaction. The GC is therefore essential for the clearance of invading pathogens and for vaccine responses.

Germinal center initiation is dependent on the coordinated activation and migration of both conventional follicular B cells and CD4 T cells. Differentiation of Tfh and GC B cells is a multistep process that relies on several factors, including T: B interaction, coreceptor engagement, and cytokine signals [36, 37]. Tfh cells derive from naive CD4 cells that bear relatively high‐affinity T‐cell receptors (TCR) for antigen and are dependent on strong TCR signaling for differentiation [38, 39]. Signaling via interleukin (IL)‐6, IL‐21, and the coreceptor inducible co‐stimulating ligand (ICOSL) further reinforces the Tfh transcriptional program through upregulation of the lineage‐defining transcription factor B‐cell lymphoma 6 (BCL6) [40, 41, 42, 43, 44]. B‐cell activation is dependent on B‐cell receptor cross‐linking for the initial activation of the cell [45]. Activation of pre‐Tfh T cells induces migration to the border between the B‐cell and T‐cell zones through downregulation of migration markers such as chemokine‐receptor type 7 (CCR7) and P‐selectin glycoprotein ligand 1 (PSGL1) along with an increase in the chemokine receptor C‐X‐C motif chemokine receptor 5 (CXCR5). The net effect of this altered surface receptor expression is to reduce the attraction to the high levels of CC chemokine ligand (CCL)19, and CCL21 expressed in the T‐cell zone and increase movement toward the C‐X‐C ligand (CXCL)13‐rich B‐cell follicle [46, 47]. Similarly, B cells alter the expression of migratory markers, increasing CCR7 after activation in order to direct migration to the T‐cell/B‐cell border [48].

Initial T‐cell and B‐cell interaction occur at the T:B border; here, B cells present antigen captured via the BCR on MHC‐II and solicit help from activated T cells. Strong interaction through TCR:MHC‐II, signaling through IL‐21, and co‐stimulation via CD40:CD40L and inducible T‐cell co‐stimulator (ICOS): ICOSL lead to differentiation of the B‐cell population into short‐lived plasmablasts, whereas intermediate or weak engagement leads to GC recruitment or memory B‐cell differentiation, respectively [49, 50, 51]. The majority of B‐cell receptor class switching occurs at this time and is likely induced by these initial T:B‐cell engagements [52]. After these early interactions, Tfh cells migrate into the follicle followed by the activated B cells, which rapidly proliferate and displace the resident naive B cells, creating the follicular mantle. Early GCs can be identified within the B‐cell follicle by day 4 postimmunization, and GCs reach peak size by day 7 after immunization [2, 53, 54]. The GC consists of two distinct zones termed light zone (LZ) and dark zone (DZ). The DZ consists of DZ GC B cells that are proliferating and undergoing SHM through the expression of activation‐induced deaminase (AID) [55, 56]. Following the completion of proliferation in the DZ, these B cells will downregulate the chemokine receptor CXCR4 and upregulate CXCR5 leading to DZ to LZ migration toward the CXCL13‐rich LZ [57].

**Fig. 2:**
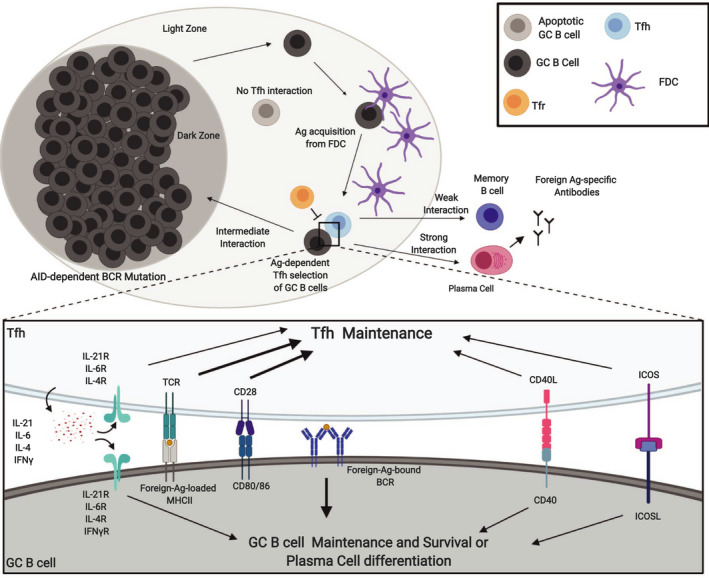
Overview of the germinal center response. Germinal center B cells (GC B cells) in the dark zone (DZ) actively mutate BCRs through AID expression. After undergoing multiple rounds of proliferation and mutation, DZ GC B cells will migrate to the light zone (LZ). Mutations that increase the antigen affinity of BCRs allow B cells bearing those receptors to gather more antigens from follicular dendritic cells (FDCs) and, in turn, present more antigens on MHC‐II. Follicular T helper (Tfh) cells in the LZ then select GC B cells based on MHC‐II antigen expression and may lead to several outcomes depending on the strength of the interaction. Weak or strong Tfh:GC B‐cell interaction will drive differentiation to memory B cells or plasma cells, respectively, while intermediate interaction induces GC B cells to return to the DZ and restart the process of BCR mutation. No interaction results in apoptosis of the B cell after approximately three days. The cell interaction itself depends on several B‐cell and T‐cell‐derived signals. Tfh cells depend on T‐cell receptor (TCR) engagement of histocompatibility complex II (MHC‐II), autocrine signaling of cytokine, and engagement of coreceptors such as CD28, CD40L, and ICOS. Similarly, the GC B cells depend on the cytokine produced by Tfh cells, CD40, and ICOSL as well as BCR engagement. Similar signals are involved in the differentiation of both Tfh and GC B cells early in the response. Overtime, high‐affinity B cells predominate through increased survival over lower affinity B cells in the GC; plasma cells are eventually derived from the high‐affinity B‐cell pool due to strong BCR signaling upon antigen engagement followed by strong Tfh interaction. Abbreviations: activation‐induced deaminase (AID), antigen (Ag), T follicular regulatory cells (Tfrs), interleukin (IL). Created with BioRender.

Light zone cell populations include Tfh cells, follicular dendritic cells (FDCs), and LZ GC B cells. FDCs are critical for the maintenance of the GC reaction through processing and retaining antigen and as the primary source for CXCL13 [58, 59]. It is in the LZ that B cells are selected for increased affinity. B cells that have acquired a mutation or mutations conferring higher affinity for antigen will gather more antigen from FDCs, bear more antigen‐loaded MHC‐II, and can more efficiently solicit help from Tfh cells through TCR:MHC‐II interaction. To facilitate this interaction, both GC B cells and Tfh cells within the LZ are highly motile in order to increase the probability of cognate antigen encounters. Upon cognate encounter, T‐cell migration and B‐cell migration are temporarily slowed resulting in Ca^2+^ influx in Tfh cells, expression of IL‐21, and IL‐4 by Tfh cells and coreceptor engagement of ICOS:ICOSL and CD40:CD40L in both cell types [60]. Together, these signals promote the survival of the antigen‐presenting B cell within the GC. Tight control over the formation or persistence of autoreactive Tfh cells as well as the numbers and effector functions of non‐self‐restricted Tfhs is thus critical for the proper regulation of GC B cells and is necessary to prevent the inadvertent selection of autoreactive B cells. Within GCs, Tfh and GC B‐cell interaction strength determine the fate of GC B cells; high strength interaction can induce PC differentiation, while intermediate or lower affinity will induce GC B cell return to the DZ or cause B‐cell memory differentiation, respectively. Over time, this reaction produces a steady increase in BCR affinity through increased survival and proliferation of the highest affinity B cells and eventually differentiation of long‐lived plasma cells that produce effector antibodies toward pathogens or vaccine components (Fig. 2).

Several groups have demonstrated that control of GC responses is critically dependent on follicular regulatory T cells (Tfrs) [61, 62, 63]. These cells display a unique transcription factor and surface receptor pattern that includes BCL6 and FoxP3 co‐expression as well as high expression of CXCR5, ICOS, CTLA‐4, and PD‐1. This combination of Treg and Tfh proteins allows these cells to gain access and exert a suppressive function within the GC. Tfrs were initially thought to be derived exclusively from the natural Treg population, although at least one recent study suggests that these cells may also be derived from naive CD4 cells. Of note, a significant difference in the TCR repertoire of Tfh versus Tfr cells has been documented [64]. Signaling by IL‐2 is mandatory to induce proliferation of early Tfrs, while IL‐21 inhibits proliferation and contributes to the final maturation of these cells [65]. How Tfr cells control the GC reaction is still incompletely understood. Using an *in vitro* co‐culture system, Sage *et al*. [66] have demonstrated that Tfr cells inhibit specific effector gene expression in both Tfh and GC B cells, while IL‐21 signaling overcomes this inhibition. Specific proteins and mechanisms for Tfr function are so far unclear; only CTLA‐4 has been shown to be critical for the suppressive function of these cells as loss of CTLA‐4 results in increased Tfh, Tfr, and GC B‐cell populations [67].

## Systemic lupus erythematosus (SLE)

Autoimmune diseases, including SLE, are thought to be the result of a complex interplay of genetic susceptibility, epigenetic changes, cytokine production and signaling, immunogenic triggers such as infectious agents, and the presence of immune‐suppressing or immune‐activating sex hormones. SLE is an autoimmune disease characterized by autoreactive antibody formation and dysregulated GCs, which primarily affects women at a ratio of 9:1 [68]. All organs may be affected by the disease, but skin, heart, lungs, and kidneys are most commonly involved, with the most severe forms of disease manifesting in glomerulonephritis, and tissue damage resulting in kidney failure. Autoantibodies directed against double‐stranded DNA (anti‐dsDNA) or antinuclear antibodies (ANA) are common. Tissue damage is largely mediated by autoantibody complex formation, which is primarily caused by the inability to clear autoantibody complexes. Polymorphisms in Fc receptors and low Fc receptor expression are known to contribute to reduced autoantibody and antigen complex clearance [69, 70]. Complex formation is also associated with the activation of complement leading to further inflammation, immune cell infiltration into affected tissues, and disease exacerbation [71, 72].

Autoreactive T cells play a significant role in SLE development through direct selection of self‐reactive B cells. In mouse, models of disease autoreactive T cells seem to arise through failures in peripheral tolerance [73, 74, 75, 76]. A broad range of immune cell defects has been observed in patients with SLE including preclustered TCR lipid rafts that contribute to altered signaling of T cells [77]. T cells from patients with SLE are often found to have reduced IL‐2 expression, which may result in more Tfh cells due to reduced differentiation into other T helper subsets that rely on IL‐2 (i.e., Th1) and lower Treg numbers. Lower IL‐2 may also contribute to reduced activation‐induced cell death, further allowing survival of autoreactive T cells [78]. Reduced IL‐2 expression can be due to the substitution of the TCRζ chain with FcγR [79, 80]. Additionally, Juang et al. demonstrated that reduced IL‐2 could be caused by autoantibodies directed against CD3. They show that stimulated healthy T cells treated with the IgG serum fraction from SLE patients caused a reduction in IL‐2 expression by T cells from healthy patients, through increased expression of the IL‐2 suppressor cAMP response element modulator (CREM) indicating some level of TCR interference via IgG:CD3 binding [81]. Similarly, SLE patients often develop cytopenias such as thrombocytopenia [82]. Increased circulating Tfh cells and higher IL‐21 expression have been linked to SLE disease activity in humans and disease induction, including spontaneous GC formation, in various mouse models [83, 84, 85]. Furthermore, metabolic defects can intrinsically influence autoreactive T cells and have been recently identified in rheumatoid arthritis and SLE [86, 87, 88, 89, 90, 91, 92, 93].

## Nuclear receptor regulation of GC responses and autoimmunity

**Fig. 3:**
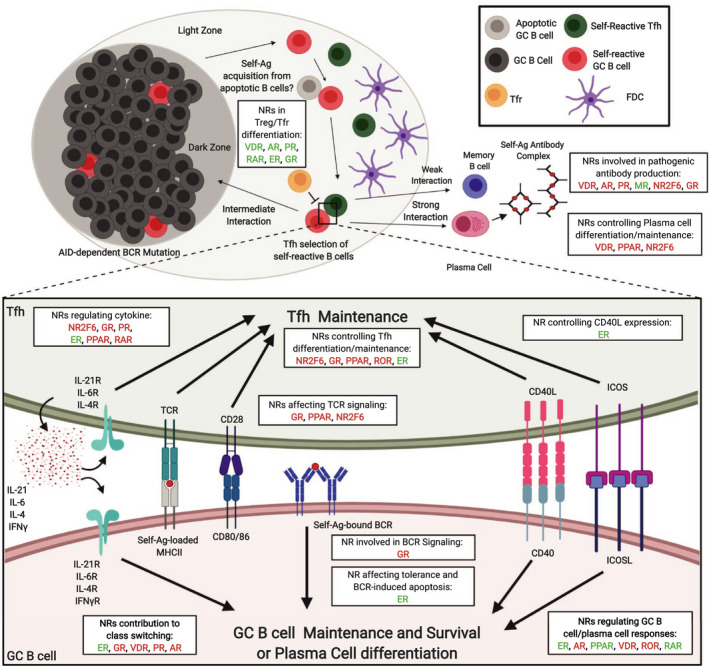
Schematic illustration of activating and inhibitory roles of nuclear receptors within the autoimmune germinal center. Self‐reactive germinal center B cells (GC B cells) may be produced through the random action of somatic hypermutation (SHM)**,** or alternatively, can avoid early negative selection through increased ER activity. GC B cells likely capture self‐antigen within the GC, possibly from the large number of apoptotic B cells. While autoreactive follicular T helper (Tfh) cells may persist through peripheral tolerance escape possibly due to lower interleukin (IL)‐2 expression. Increased numbers of Tfh cells may reduce the limitedness of Tfh help and can lead to inadvertent selection of self‐reactive B cells (not shown). Tfh numbers can be controlled by several NRs including NR2F6 and PPAR, and higher Tfh numbers can be achieved by increased survival (NR2F6) or increased differentiation of naïve cells to the Tfh lineage (PPAR). NRs may also control cytokine expression in Tfh cells and in this way contribute to inadvertent selection of self‐reactive GC B cells (i.e., NR2F6, GR, and PPAR). T regulatory (Treg) and follicular T regulatory (Tfr) cell differentiation may be affected by NRs including VDR, RAR, and AR. NRs such as AR and VDR can control the production of self‐reactive antibodies, while others such as MR may reduce class switching to more pathogenic isotypes. Finally, NRs such as VDR can reduce differentiation of B cells into plasma cells and thus may reduce autoantibody production. NRs are labeled to indicate either promotion (green) or suppression (red) for each indicated phenomenon in autoreactive GCs. Abbreviations: estrogen receptor (ER), mineral corticoid receptor (MR), androgen receptor (AR), progesterone receptor (PR), peroxisome proliferator of activated receptor (PPAR), nuclear receptor subfamily 2 group F member 6 (NR2F6), all‐trans retinoic acid receptor (RAR), retinoic acid receptor‐related orphan receptor (ROR), vitamin D receptor (VDR), antigen (Ag). Created with BioRender.

Despite the considerable evidence linking nuclear receptors to general immune function or dysfunction, there is little research on molecular mechanisms for this family of receptors within Tfh, GC B cells or supporting cells such as FDCs. However, several NRs have been shown to play a role in the proper function of these cellular subsets (Fig. 3, see also Table 1), and analysis of sex hormone levels in combination with the status of immunocompetence has already been proposed for vaccine study participants in clinical trials and during vaccination per se [94, 95, 96]. In the remaining sections of this review, we focus on the effect of specific NRs in T and B cells with a special emphasis on GC responses and SLE.

**Table 1:** Overview of nuclear hormone receptors and their role in SLE. NRs in red are generally considered to be detrimental for disease occurrence and progression, whereas NRs in blue are considered to be protective and beneficial. In essence, treatment should include drugs with agonistic behavior for the blue NRs, and antagonistic effects for the red NRs. The table is based on references within the main text. Only naturally occurring ligands are listed. CSR, class‐switch recombination; DC, dendritic cell; DHEA, dehydroepiandrosterone; DHT, dihydrotestosterone; GC, germinal center; RA, retinoic acid; SLE, systemic lupus erythematosus; TCR, T‐cell receptor; TLR, Toll‐like receptor.

Subfamily	Member(s)	Ligand(s)	Role in immune cells and SLE
Steroid hormone receptors			
Estrogen receptor (ER)	ERα (NR3A1, ESR1)	17β‐estradiol, estriol, estrone	*Human:* ER signaling effects are dose‐dependent, with 9 times more prevalence in women with SLE
	ERβ (NR3A2, ESR2)	17β‐estradiol, estrone	Increased ER‐mediated expression of CD40L and IL‐21
			More ER signaling and coreceptor expression in SLE patient T cells through decreased methylation of the CD40L gene
			*Rodent:* ER binding to *Ifng* promoter; more IFNγR signaling on GC B cells; ERα suppresses Tfh cell function
Glucocorticoid receptor (GR)	GR (NR3C1)	Aldosterone, progesterone, glucocorticoids: corticosterone, cortisol, deoxycorticosterone	*Human:* GR induces BLIMP‐1 and IL‐10 production in Tregs and reduces Tfh cell numbers
			Gender bias; GR downregulates X‐chromosomal expression of TLR7, and as a result inflammatory signaling
			GR induces Tfh apoptosis in SLE patients
			*Rodent:* GR‐induced signaling leads to reduced T‐cell activation
			Important for elimination of low‐affinity TCR T cells
			GRs downmodulate co‐stimulatory molecule expression by DCs
			Important for immunoglobulin class switching
Mineralocorticoid receptor (MR)	MR (NR3C2)	Aldosterone, progesterone, glucocorticoids: corticosterone, cortisol, deoxycorticosterone	*Human:* MR enhances homing to secondary lymphoid organs and immune cell activation.
			Enhanced MR signaling is associated with hyperkalemia in blood
			*Rodent:* MR contributes to murine renal nephritis, with enhanced proteinuria and serum autoantibodies
			Regulates circadian rhythm, blood potassium, and salt levels
Progesterone receptor (PR)	PGR (NR3C3)	Progesterone	*Human:* Progesterone reduces pro‐inflammatory cytokine production in T cells
			Increase in Treg differentiation
			Reduction in co‐stimulatory molecules and pro‐inflammatory cytokines by DCs
			*Rodent:* Reduction in B‐cell CSR
			Decreased T‐cell‐dependent antibody responses
Androgen receptor (AR)	AR (NR3C4)	DHT, DHEA, testosterone, androstenedione	*Human:* High serum DHT levels are associated with less mature B cells
			Female SLE patients have generally lower androgen levels
			*Rodent:* Reduction in co‐stimulatory molecules and MHC expression on DCs
			AR inhibits B‐cell lymphopoiesis and class switching to pathogenic IgG
			Enhance negative selection of autoreactive T cells and promote tolerance in thymus
			Enhance serum complement components that aid in clearance of immune complexes
Nonsteroid hormone receptors			
Peroxisome proliferator‐activated receptors (PPAR)	PPARα (NR1C1)	Leukotriene B4, fatty acids, eicosanoids	*Human:* PPARα major player in liver lipid metabolism. SLE patients have higher incidence of early‐onset atherosclerosis
			*Rodent:* PPARα represses NF‐κB and c‐Jun in T cells, leading to lower production of Th1‐mediated cytokines
	PPARβ/δ (NR1C2)	Fatty acids, eicosanoids	Activated PPARβ/δ increases lipogenesis in liver and skeletal muscle cells
			*Rodent:* PPARβ/δ increase dead cell removal by macrophages
			PPARβ/δ improves vascular function and protects against kidney damage
	PPARγ (NR1C3)	Fatty acids, prostaglandin J2, eicosanoids	*Human:* Activation of PPARγ leads to enhanced B‐cell proliferation and antibody production
			PPARγ in SLE macrophages represses CD40/CD40L pathway
			*Rodent:* PPARγ inhibits T‐cell activation and Tfh differentiation
Retinoic acid receptor (RAR)	RARα (NR1B1)	Vitamin A (RA)	*Human:* Vitamin A reduced in SLE patients
	RARβ (NR1B2)	Vitamin A (RA)	RA in gut leads to more Tregs and suppression of autoimmunity
	RARγ (NR1B3)	Vitamin A (RA)	Treatment with RA reduces nephritis pathology
			*Rodent:* RA inhibits pro‐inflammatory cytokine signaling
			RA important in protective IgA production by gut B cells
Vitamin D receptor (VDR)	VDR (NR1I1)	Vitamin D	*Human*: VDR signaling reduces B‐cell proliferation and induced apoptosis Differentiation of plasma cells is reduced as well as class‐switched memory B cells
			SLE patients have reduced levels of Vitamin D
			*Rodent:* Promotes Th2 and Tregs over Th1 and Th17 cell differentiation
RAR‐related orphan receptor (ROR)	RORα (NR1F1)	Orphan (oxysterols^a^)	*Human:* ROR genes increased in human Tregs
	RORγ (NR1F3)	Orphan (oxysterols^a^)	RORs mediated IL‐17 overexpression in human SLE is linked to increased disease severity
			*Rodent:* ROR NRs are important for IgA‐producing memory B‐cell homeostasis
			RORγt lineage, defining for Th17 subset, suppress Tfh differentiation
			RORs protective against spontaneous GC formation
COUP/EAR	NR2F6 (EAR‐2, COUP‐TFIII)	Orphan	*Rodent:* NR2F6 binds to promoter regions and suppresses expression of IL‐21, IFNγ, IL‐2 and IL‐17
			NR2F6 suppresses accumulation of GC B cells, plasma cells, and Tfh cells
			Aged NR2F6‐deficient mice have SLE‐like symptoms such as auto‐antibodies

^a^ The natural occurring ligands are still unknown, although recently oxysterols have been proposed.

## Steroid hormone receptors

Steroid hormone receptors are divided into two subfamilies, the first being the estrogen receptor family which is comprised of estrogen receptor‐α (ERα; NR3A1, ESR1) and estrogen receptor‐β (ERβ; NR3A2, ESR2). The remaining receptors belong to the ketosteroid receptor family, which include the glucocorticoid receptor (GR; NR3C1), mineralocorticoid receptor (MR; NR3C2), progesterone receptor (PR; NR3C3, PGR), and the androgen receptor (AR; NR3C4, AR).

### The estrogen receptor family (ER; NR3A)

Estrogen is mainly synthesized by mammalian ovaries but can also be produced in cells expressing the enzyme aromatase and is primarily secreted into the blood. Due to its lipophilic nature, it passes freely through cell membranes, where it binds to the estrogen receptor (ER) [97]. ER exerts its function through multiple mechanisms, termed ‘genomic’, ‘tethered’, ‘nongenomic’, and ‘ligand‐independent’ [97]. Understanding the biological effects of both estrogen receptors, ERα and ERβ, within the immune system, are especially crucial for unraveling the known gender‐dependent differences during pathogen encounter and autoimmune diseases. In contrast to the other steroid hormone receptors, estrogen is known to promote immune responses, especially humoral immunity.

ER signaling plays an important role in B‐cell development, activation, and function. Signaling via ER has been shown to prevent negative selection of autoreactive B cells at multiple stages of development and to alter BCR signaling thresholds as well as activation‐induced apoptosis [98, 99]. Following influenza virus infection, female mice demonstrated a better virus‐specific antibody response, with higher serum levels in the IgG2b subclass [10]. In B cells, estrogen enhances the expression of AID and induces class switch recombination (CSR) [11]. Mechanistically, ERα directly binds to several regulatory regions such as Sµ and Eµ, and hypersensitivity sites HS1, HS2, and HS4 of the 3′ regulatory region within the immunoglobulin heavy‐chain gene sequence and, through binding to these sites, influences class switching [10].

Stimulated T cells from both mice and humans express more IFNγ in the presence of estrogen likely through direct binding of ER to the *Ifng* promoter region [12]. In mice, increased IFNγ signaling is known to contribute to SLE development through direct signaling to GC B cells via the IFNγ receptor [13]. ER function seems to be dependent on the type of inflammation or immune response. For example, ERα signaling appears to promote Th1 and Th17 accumulation while decreasing FoxP3 expression of CD4 T cells in a mouse model of colitis [14]. However, in human cervical cancer, ERα signaling can drive FoxP3 expression in infiltrating Tregs [15]. Similarly, ER signaling has been shown to increase Treg numbers during pregnancy [16].

Kim *et al*. identified ERα loss within the CD4 population of non‐SLE prone mice as a driver of SLE‐like disease. This observation seemed to be due to a specific function for ERα in the suppression of Tfh cells as ERα‐deficient mice had larger Tfh populations when compared to wild‐type, and administration of 17β‐estradiol reduced Tfh numbers in wild‐type mice [17]. Interestingly, in mice that are susceptible to SLE‐like disease, administration of 17β‐estradiol increases disease severity, while ovariectomy reduces disease progression [18]. Conflicting data exist regarding the loss of ERα in male mice. Loss of ERα in SLE‐prone NZB/NZW F1 mice results in reduced amounts of anti‐dsDNA antibodies and increased survival of both male and female mice [19]. Svenson *et al*. [110] observed improved survival of female mice only with ER deficiency in two separate mouse models of SLE with no change in anti‐dsDNA production. This suggests that, in mice not susceptible to SLE, ERα may have a suppressive effect on disease, but its function may be defective or otherwise altered in SLE.

Due to the sex bias observed in SLE, the role of ERs has been an area of extensive study. Estrogen is known to be a risk factor for SLE development, as illustrated by the increased risk for SLE in women during childbearing years and a subsequent decrease in risk after menopause [111]. Furthermore, estrogen supplementation has been documented to induce SLE activity [112]. However, prepubescent children, postmenopausal women, and men can develop SLE [111]. Several genes on the X chromosome have been linked to SLE, including the immune‐relevant receptors CD40L and CXCR3, together with microRNAs including miR‐98 and miR‐188‐3p [113, 114]. Subsequent hypomethylation within gene regulatory regions has been correlated to overexpression of numerous other immune‐relevant non‐X‐linked genes, such as IL‐10, IL‐4, IL‐6, protein phosphatase 2a (PP2Acα), CD11a, STAT1, and MX1 [115]. Similarly, hypomethylation has been linked to inefficient X chromosome inactivation in SLE patients [114]. A proof‐of‐concept study in mice with defects in ERK signaling and, as a result, reduced DNA methylation in CD4 T cells resulted in overexpression of several X‐linked genes such as *Cd11a*, *Cd70*, and *Cd40l* and SLE development; further estrogen addition exacerbated the disease. In this mouse model, only female mice developed SLE, and the authors link this to a requirement for two X chromosomes consistent with dysregulated X chromosome inactivation [116]. Demethylation of the gene encoding ERα has also been linked to overexpression of this NR and is associated with increased expression of ER‐sensitive genes [117]. Wu *et al*. observed increased ERα protein and 17β‐estradiol signaling in CD4 T cells from SLE patients that resulted in global DNA hypomethylation through downregulation of DNA methyltransferase 1 (DNMT1). Treatment of PBMCs with an ER antagonist ICI182780 increased DNA methylation [118]. Altered methylation status may explain why ER signaling can upregulate CD40L on PBMCs from lupus patients, and the antagonist (ICI182780) can prevent overexpression of CD40L [119]. ER‐mediated downregulation of DNMT1 may also contribute to the direct X‐chromosome effects observed in SLE, as well as non‐X‐linked immune gene overexpression in SLE patients through reduced DNA methylation.

There is some evidence for a nongenomic signaling role for ER in SLE; treatment of SLE patient‐derived peripheral blood mononuclear cells (PBMCs) with 17β‐estradiol induced a mitogen‐activated protein kinase (MAPK)‐dependent increase in IL‐21 expression in these cells [120]. Others have observed an increase in CD40L and also in calcineurin expression after ERα and ERβ agonist treatment of PBMCs from SLE patients [121], suggesting that defects in ER signal transduction may also contribute to SLE in both mice and humans. The exact defect in ER signaling may vary depending on mouse strain and genetic background.

Taken together, ER signaling is a known risk factor for SLE development; this is due in part to its roles in promoting humoral immune responses, reducing methylation of DNA leading to aberrant gene expression and potentially through altered nongenomic signaling pathway in SLE patients.

### Glucocorticoid receptor (GR; NR3C1)

Glucocorticoids bind to the GR and contribute to diverse biological processes, including glucose metabolism, stress, and immune responses. The GR is expressed in most cells of the body and is a multitasking transcription factor, changing its role and function from anti‐inflammatory effects to potential pro‐inflammatory actions; a key component of the mechanism of action of glucocorticoids involves interference with the activity of other transcription factors and signaling molecules (reviewed in Ref. [122]). Rapid nongenomic mechanisms of glucocorticoid signaling have also been reported.

Glucocorticoids have been used to treat a broad range of inflammatory and autoimmune disorders, including SLE, since the initial discovery of their potent anti‐inflammatory properties by Hench and colleagues in 1948 [123]. GR signaling in the immune system is complex and may depend on several factors, including the particular glucocorticoids present and the relative level of these hormones. Glucocorticoid effects are often observed to be biphasic in that higher doses may differently affect a gene or function relative to lower doses [124]. GRs and glucocorticoids have a significant effect on cells of the immune system including T and B cells [122].

In mice, the GR is present in all B‐cell developmental subsets; the synthetic and potent glucocorticoid, dexamethasone, reduces cell viability in all B‐cell subsets, both *ex* and *in vivo* [125]. Franco *et al*. [126] found that glucocorticoids impair upstream B‐cell receptor and Toll‐like receptor (TLR)‐7 signaling, reduces transcriptional output from the three immunoglobulin loci, and promotes significant upregulation of the genes encoding the immunomodulatory cytokine IL‐10 and the plasma cell differentiation factor BLIMP‐1. Work from Fleshner *et al*. demonstrated that adrenalectomy or GR blockade in rats is associated with defective IgM and IgG responses to T‐cell‐dependent antigen; this was linked to roles in class switching from IgM to IgG2a, suggesting that basal glucocorticoid signaling is necessary for regular class switching [127]. Importantly, the glucocorticoid‐mediated downregulation of TLR7 expression and functional impairment of TLR7 signaling have implications on gender differences in the incidence of autoimmune diseases as human TLR7 resides in the X chromosome and escapes X inactivation in human B cells from SLE patients [128].

Mice in which the GR has been deleted from T cells are immune‐deficient due to a skewed TCR repertoire toward those with a low affinity for MHC [129]. Glucocorticoids also reduce signaling in mature T cells through suppression of NF‐κB, NFAT, and AP‐1 activation [130]. GRs can reduce the expression and activation of several essential mediators of TCR signaling including Lck, Fyn, and ITK [131]. Glucocorticoids also upregulate BLIMP1 expression in human CD4 T cells [126]. Increased BLIMP‐1 expression, in turn, promotes the function of Tregs, directly induces IL‐10 expression, and can reduce Tfh differentiation [132, 133]. Additionally, DCs downregulate co‐stimulatory receptors, and pro‐inflammatory cytokines in response to GR signaling, reducing T‐cell activation [134, 135].

Glucocorticoid receptor regulate the diurnal oscillation of T cells through regulation of CXCR4 and IL‐7R expression, with lymphocytes accumulating in lymphoid tissues overnight and moving to the blood during the day. In an elegant study by Shimba et al., immunization at night increased GC responses, and this effect was lost with GR deficiency in the CD4 population. Loss of GR also affected Tfh expression of IL‐4 and IL‐13. Finally, Peyer’s patch GCs were unaffected by diurnal cycles; however, loss of GR reduced both Tfh and GC B cells at this location [136].

The beneficial effect of glucocorticoids on SLE is likely due to multiple modes of action, including the effects mentioned above on T cells and DCs. T‐cell anti‐inflammatory activity by glucocorticoid treatment is reduced due to a combination of increased migration to and impaired release from lymphoid tissues [137, 138]. GRs and glucocorticoids may control B‐cell numbers directly through regulation of the target gene GILZ, and global loss of this gene can induce SLE‐like disease in mice [139]. Otherwise, B‐cell control by GRs is thought to be similar to T‐cell control as well as through effects on survival and TLR‐7 signaling mentioned above. Loss of GR in the Treg population results in an inability of these cells to control ANA formation. These phenomena were linked to GR‐mediated suppression of Th1‐like characteristics, including IFNγ expression by Tregs [140]. Finally, treatment with glucocorticoids has been shown to induce apoptosis of circulating Tfh cells in patients with SLE, both *in vitro* and *in vivo* [141].

### Mineralocorticoid receptor (MR; NR3C2)

Although best known as the ‘aldosterone receptor’ that regulates electrolyte and fluid homeostasis in the distal nephron and other epithelial tissues, the mineralocorticoid receptor (MR) has received increasing attention as a driver of cardiovascular and renal fibrosis. While the primary mineralocorticoid ligand for the MR is aldosterone, the MR can also bind and respond to glucocorticoids [142]. MR is expressed in cells of the immune system, where it responds to stimulation or antagonism, controlling immune cell function. Aldosterone has been associated with pro‐inflammatory immune effects, such as the release of cytokines that promote oxidative stress, and fibrosis, and exacerbates multiple sclerosis in murine models [143, 144].

MR signaling in human T cells regulates the redistribution of T‐cell subsets to lymph nodes, through effects on CD62L, CCR7, and CXCR4 [145]. MR activation during periods of high aldosterone and low cortisol levels (e.g., nocturnal sleep) seems to provide the optimal endocrine milieu for facilitated homing of naive T cells to the lymph nodes. Nocturnal sleep has been consistently found to benefit the formation of an antigen‐specific immune response, using experimental vaccination in humans [146, 147]. Facilitation of T‐cell homing following sleep‐dependent aldosterone release might thus substantially contribute to the well‐known role of sleep in supporting antibody responses [145].

Aldosterone receptor blockade is safe and well‐tolerated in progressive murine lupus nephritis, results in decreased levels of clinical proteinuria and lower serum levels of autoantibodies, and is coupled with decreased kidney damage. MR appears to modulate inflammatory changes during the progression of glomerulonephritis and may also have a previously undescribed role in attenuating apoptosis [148].

### The progesterone receptor (PR; NR3C3, PGR) and the androgen receptor (AR; NR3C4, AR)

Progesterone and androgens are commonly thought of as sex hormones but are known to have an overall suppressive function on immune cells via signaling through the progesterone receptor (PR) and androgen receptor (AR), respectively [149, 150, 151].

Interestingly, progesterone seems to reduce the risk of SLE by counteracting the effects of estrogen, which suggests that the balance between estrogen and progesterone can determine disease manifestation [152] and is especially interesting concerning pregnancy‐related remission of the autoimmune diseases RA and MS [153].

The detailed functional role of both androgens and progesterone in B and T cells as well as cellular and molecular targets in autoimmune diseases such as SLE or rheumatoid arthritis has recently been reviewed in detail and will therefore not be covered in detail in the current review [150, 152, 153]

## Nonsteroidal nuclear receptors

### Peroxisome proliferator‐activated receptors (PPARs; NR1C)

The PPAR family comprises three distinct receptors (PPARα, PPARγ, and PPARδ) that recognize fatty acid metabolites as ligands. These receptors regulate a broad range of biological processes including angiogenesis, glial cell maturation, inflammation, and glucose and lipid metabolism [154, 155]. PPARs, particularly PPARγ, have been linked to both T‐cell function and autoimmunity [156]. Agonists for PPARγ (NR1C3) have proven beneficial in animal models of multiple sclerosis, asthma, and rheumatoid arthritis and human patients with colitis [157, 158, 159, 160].

Activated B cells upregulate PPARγ expression. Nanomolar levels of synthetic PPARγ ligands such as rosiglitazone enhance human B‐cell proliferation and induce plasma cell differentiation as well as antibody production, whereas the addition of GW9662, a specific PPARγ antagonist, abolishes these effects [161]. Furthermore, combinatorial activation of both PPARγ and its binding partner RXR results in additive effects via enhancing Cox‐2 and BLIMP‐1 expression, suggesting that low doses of PPARγ/RXRα ligands could be used as an adjuvant to stimulate antibody production [161].

Conditional deletion of PPARγ in the mouse CD4 T‐cell compartment leads to general CD4 hyper‐reactivity during *in vitro* TCR stimulation, characterized by increased cytokine expression and proliferation, under Th1‐, Th2‐, Th17‐, and Th9‐polarizing conditions [162]. Additionally, PPARγ‐deficient CD4 cells exhibit reduced expression of negative regulators of NF‐κB signaling, including Sirt1 and IκBα. An increase in AKT and ERK phosphorylation was observed in addition to reduced Foxo1. *In vivo,* PPARγ‐deficient mice develop exaggerated Tfh populations after immunization with sheep red blood cells (SRBCs) as well as spontaneous GC formation, increased plasma cell number, and elevated titers of anti‐dsDNA antibodies [162]. These observations are likely linked to increased AKT signaling driving the loss of Foxo1, which is a crucial suppressor of BCL6 expression and Tfh differentiation [40, 163, 164].

The PPARγ agonists pioglitazone and rosiglitazone have been tested in the MRL*^lpr^* mouse model of SLE for effects on disease activity. In both cases, these compounds reduced disease severity when used early before disease onset but had minimal effect after disease manifestation. Both agonists significantly improve SLE‐related atherosclerosis [165, 166]. Furthermore, two separate groups utilized the NZB/W F1 cross SLE mouse model to investigate the protective potential of PPARγ agonists rosiglitazone and pioglitazone. Both groups found a significant protective benefit on renal disease severity when these drugs were administered early in the course of disease [167, 168]. Targeting of PPARγ in SLE thus appears to have some benefit on symptoms of SLE but no significant impact on disease progression in mice. This may indicate that PPARγ is more critical for the early activation and differentiation of Tfh cells compared with reactivation of memory Tfh cells that are presumably driving SLE at later time points. Memory Tfh cells are an understudied population, and a role for PPARγ in these cells is currently unknown.

Another study observed a marked decrease in the activation and proliferation of human PBMCs derived from SLE patients when treated with pioglitazone [169].

Interestingly, the suppressive effects of PPARγ on Tfh cells appear to be also dependent on the estrogen receptor. Park et al. demonstrated that treatment with the PPARγ agonist pioglitazone reduces Tfh accumulation in female but not male mice. Intriguingly, treatment of male mice with both pioglitazone and 17β‐estradiol, a natural ligand for both ERα and ERβ, reduced the number of Tfh cells, whereas treatment with either substance alone had no significant effect. Pioglitazone also had differential effects on female mice, being more effective during the estrous phase of the menstrual cycle when estrogen levels are highest [170, 171]. Similarly, differential effects of pioglitazone were observed in the differentiation of Th1, Th2, and Th17 cells a phenomenon that was also linked to estrogen availability [171, 172]. The ability of these agonists to prevent SLE in humans is unlikely given the relatively minor effect these drugs had on mouse models but has not yet been investigated. However, pioglitazone is currently in phase II clinical trials aimed at testing its ability to ameliorate the vascular complications associated with SLE [173].

### Retinoic acid receptor (RAR; NR1B)

The RAR family consists of three members, RARα, RARβ, and RARγ. Together, these receptors mediate the response to the vitamin A metabolite, retinoic acid (RA), and are often paired as a heterodimer with RXR. All immune cells are responsive to RA signaling, and DCs and macrophages can modify the RA metabolite retinol to the more active signaling form, namely all‐*trans*‐RA [174]. In T cells and B cells, as well as DCs, RA signaling mediates migration to gut tissue through upregulation of the gut homing integrin α4β7. RA is also critically important for the proper functioning of T cells and B cells, as well as responses to vaccination [174].

Signaling via RARs is essential for both B‐cell development and function. The presence of RA is known to accelerate the differentiation of B cells from lymphoid progenitors. It does so by promoting crucial transcription factors involved in B‐cell lymphopoiesis including PAX5 and early B‐cell factor (EBF)‐1. In the periphery, RA signaling promotes plasma cell differentiation through the upregulation of interferon regulatory factor (IRF)‐4 and BLIMP1 [175].

RA has a prominent role in the migration and antibody output of innate B cells while not affecting follicular (B2) cells to the same extent. For example, loss of RARα function through the expression of a dominant‐negative RARα (dnRARα) results in altered IgM and IgG3 expression and lower levels of IgA from innate B cells [176]. Interestingly, only B1b innate B cells upregulate α4β7 in response to RA signaling in the peritoneum; other innate (B1a) or follicular B cells in this location are unaffected by RA signaling [176]. RA also plays a role in IgA class switching of follicular B cells. Pantazi *et al*. demonstrated that expression of dnRARα in all B cells results in reduced IgA^+^ plasma cells in the gut. This observation was not due to reduced migration or numbers of B cells in gut Peyer’s patches (PPs) but seemed to be due to failure to class switch to IgA, as IgA^+^ GC B cells were reduced with dnRARα expression in PPs, further leading to altered microbiota and reduced responses to oral vaccination [177].

RA signaling and RARs play a critical role in T cells, in particular, in the development of gut‐tropic Tregs and can suppress the differentiation of inflammatory Th1 and Th17 cells. RARα appears to be the more critical isoform within the T‐cell subset as loss of this receptor in T cells results in altered T‐cell homeostasis similar to mice with vitamin A deficiency [178]. However, the exact function of RA on T cells appears to be context‐dependent, including the level of RA present as well as the inflammatory milieu. The addition of RA during tetanus toxin immunization results in increased Th2 differentiation [179], while interference with RARα function in CD4 cells leads to increased IL‐17 producing cells and concomitantly increases Treg differentiation [180].

Patients with SLE often exhibit reduced levels of vitamin A which can lead to reduced Treg numbers and potentially contributes to disease; treatment with RA has been shown to correct this Treg imbalance [181]. RA has also been shown to inhibit the function of several pro‐inflammatory transcriptional mediators of type I interferon signaling in mice known to contribute to SLE pathogenesis, such as Pin1, a regulator of IRF7‐TLR7/9 signaling. A similar role for Pin1 on IRF7 signaling was observed in PBMCs from SLE patients treated with RA [182]. In several mouse models of SLE, RA was able to inhibit disease initiation [182]. RA treatment has been shown to be beneficial in treating SLE nephritis in both humans and murine models [183, 184]. However, several studies investigating the effect of RA on organs affected by SLE observed mixed effects of RA treatment in the MRL^lpr^ mouse model of disease; increased disease scores in skin, brain, and lungs were detected following RA treatment; paradoxically increased lymphocyte infiltration into kidney but normalized glomeruli size was also observed [185, 186]. Thus, RARs may contribute to SLE suppression, possibly through effects on Tregs. The ability of RA to modulate disease in mouse models is not clear and warrants further investigation.

### RAR‐related orphan receptor alpha and gamma (RORα; NR1F1, RORγ;NR1F3)

Three receptors make up the retinoic acid receptor‐related orphan receptor (ROR) groups, RORα, RORβ, and RORγ. These receptors play roles in circadian rhythm, cancer, and neuron development. Additionally, RORα and RORγ play essential roles in metabolism, immunity, and autoimmunity. Alternative splicing of RORγ results in the second isoform of this receptor known as RORγt that is primarily expressed by immune cells [187]. RORγ has been recently described to bind several naturally occurring sterols [188, 189]. Synthetic ligands exist for all ROR members [190].

In B cells, RORα is crucial for the long‐term survival of IgA^+^, but not IgG2a^+^ memory B cells, as knockdown of RORα mRNA or administration of a RORα and RORγt inhibitor reduced survival of these cells. Administration of the same inhibitor also reduced BCR expression of IgA^+^ memory cells, and thus, ROR family members contribute to IgA+ memory B‐cell homeostasis [191]. Additionally, increased B‐cell proliferation, as well as spontaneous GC formation, was observed with loss of RORγt [192].

Both RORα and RORγt are essential for the differentiation of Th17 cells with RORγt being considered the lineage‐defining marker for this subset [28]. For full Tfh differentiation, these NRs must be suppressed by BCL6 [193, 194]. Loss of RORγt results in exaggerated Tfh populations overexpressing IL‐17 and IL‐21 and reduced Treg cell numbers, which may be due to partially overlapping gene profiles of Tfh and Th17 cells and shared transcription factor expression, including IRF4 and BATF, potentially causing Th17‐destined cells in the absence of RORγt to ‘default’ to the Tfh lineage. The exact cause of this observation is currently unclear. Yang and colleagues have observed higher RORC and RORA (the human RORγ and RORα genes, respectively) expression in a human subset of Treg cells that closely resemble Tfrs when compared to other Treg subsets, suggesting that these receptors may have a significant functional role in Tfr cells [195].

RORγt‐expressing Tregs can promote SLE in the pristine lupus mouse model through IL‐17 expression. Several groups have linked IL‐17 to SLE pathogenesis demonstrating that IL‐17A‐ and IL‐17F‐deficient mice fail to develop pristine‐induced SLE [196, 197, 198]. Similarly, other groups have linked Th17 cells and the expression of IL‐17 to mouse models of SLE [199, 200]. Human SLE is also often associated with increased IL‐17 in serum and IL‐17‐expressing cells (including Th17) in the periphery, and increases in IL‐17 expression have been found to correlate with SLE activity [201, 202, 203].

### Vitamin D receptor (VDR; NR1I1)

Vitamin D is an essential metabolite with significant immune functions. There are two primary sources for vitamin D, one being synthesis by the skin after exposure to ultraviolet light and the other through dietary intake. The liver hydroxylates vitamin D to 25‐(OH)D3 that can be further modified by cells expressing the hydroxylase CYP27B1 to form the biologically active 1,25‐(OH)2D3 [24]. The active form of vitamin D binds to the vitamin D receptor (VDR), which is typically found as a heterodimer with RXR, and this complex, in turn, mediates the effects of vitamin D at the cellular level. VDR is highly expressed in cells of the immune system, and its signaling has been shown to have significant effects on T cells, macrophages, dendritic cells, and B cells [24].

Human B‐cell proliferation and survival are reduced, and apoptosis is increased after treatment of PBMCs with 1,25‐(OH)2D3; differentiation of plasma cells and class‐switched memory B cells is also reduced [25]. Within the GC, VDR is differentially expressed between DZ and LZ GC B cells, with a unique cell surface expression pattern on LZ B cells. Within this compartment, VDR may contribute to the directed migration of GC B cells from DZ to LZ or may prevent excessive LZ B‐cell differentiation to plasma cells, although the exact function is currently unknown [26]. Additionally, VDR appears to be upregulated in B cells stimulated with anti‐CD40 and IL‐4, conditions that drive plasma cell differentiation *in vitro*. The functional consequence of this upregulation has yet to be defined but may be linked to a suppressive or homeostatic role of VDR in plasma cells.

T cells can be indirectly affected through the DC compartment, as 1,25‐(OH)2D3 production by DCs results in reduced DC maturation and thus fewer activated T cells [207, 208]. Vitamin D has been shown to promote the differentiation of both Th2 and Tregs while simultaneously suppressing differentiation into the pro‐inflammatory Th1 and Th17 subsets [208, 209].

Interestingly, in humans, polymorphisms in VDR are known to correlate strongly with SLE [210, 211, 212]. SLE patients often have reduced levels of vitamin D. Vitamin D supplementation was shown to increase Treg cell numbers in SLE patients but did not have a significant effect on disease symptoms. However, vitamin D treatment has shown significant benefits in patients with childhood‐onset SLE [213, 214]. Treatment of MRL^lpr^ mice with 1,25‐(OH)2D3 improved SLE symptoms, while deficiency of VDR in non‐SLE‐prone mice resulted in complement deposition within the kidney similar to mice with SLE, although with a delayed onset [215]. Thus, altered VDR signaling or decreased function may contribute to the survival or differentiation of pathogenic plasma cells. The polymorphisms associated with VDR in SLE may account for the minor effect of vitamin D supplementation on the disease. Treg induction by vitamin D may also be essential but seems to be secondary to other functions of vitamin D and VDR.

### Nuclear receptor subfamily 2 group F member 6 (NR2F6; EAR‐2, COUP‐TFIII)

NR2F6 has been shown to participate in a broad array of functions in the mammalian system, including colon homeostasis, circadian rhythms, nociception, renin expression in kidney cells, and survival and metastasis of certain cancers [216, 217, 218]. Work from our group and others have linked this receptor to immune function during cancer surveillance and autoimmunity [219, 220, 221]. In *Nr2f6*‐deficient mice, cancer outgrowth is reduced due to increased T‐cell infiltration and T‐cell effector functions, including secretion of IL‐2 and IFNγ [221, 222].

We have demonstrated that loss of *Nr2f6* exacerbates experimental autoimmune encephalomyelitis (EAE); mechanistically, NR2F6 serves as a negative regulator of *Il17* expression through DNA binding and competition for NFAT/AP‐1 transcription factor binding sites, thus reducing IL‐17 expression and pathogenesis of Th17 cells [220]. Aged *Nr2f6*‐deficient mice also develop an SLE‐like pathology, characterized by increased T‐ and B‐cell numbers and autoreactive antibodies to ANA and dsDNA [220]. Our group has recently investigated the function of NR2F6 in the GC response in more detail. *Nr2f6* deficiency resulted in increased accumulation of GC B cells, plasma cells, and to a greater extent Tfh cells in mice following immunization. We found that NR2F6 controls the expression of *Il21* through direct binding at multiple defined sites within the *Il21* locus. Lack of control of this cytokine is a significant factor in Tfh accumulation as IL‐21R blockade following immunization reduced Tfh accumulation in *Nr2f6*‐deficient mice to wild‐type levels. Interestingly, the mechanism of NR2F6 control of IL‐21 expression seems to be independent of NFAT/AP‐1 [223]. Of note, IL‐21 dysregulation and Tfh accumulation are essential drivers of mouse models of SLE and correlate with human disease; therefore, changes in IL‐21 and Tfh cells are likely a primary cause for SLE in *Nr2f6*‐deficient mice [84, 85, 224, 225].

Tfr but not Treg numbers are significantly enhanced in *Nr2f6‐*deficient mice ten days after OVA‐alum immunization, with a trend already visible on day 4 [223]. Therefore, we speculate that these Tfr cells may derive from naive CD4 T cells and not from Tregs, as has been demonstrated by the Linterman group [226]. As Tfr cell numbers are regulated by secreted cytokines such as IL‐2 and IL‐21 signals, as well as a self‐antigen presentation by B cells [65, 227], it is plausible that in addition to IL‐21 and IL‐2 that self‐antigen presentation is altered within the GC of *Nr2f6*‐deficient mice. The Tfr cell‐intrinsic role of NR2F6, therefore, needs further investigation.

## Summary and outlook

Substantial advances have been made over the past several years in our understanding of how NRs regulate immunity and germinal center responses. Recent advances have shown that Tfh cells are not only crucial during GC responses but may have a broader range of action such as promoting CD8 memory formation or as targets of tumor immune checkpoint inhibition—the future will show if NRs are also regulating these aspects of Tfh function [228, 229, 230].

Taken together, the NR family has a significant impact on cells of the immune system, and the GC response is no exception. This is highlighted by the effects of multiple NRs and their ligands on autoimmune GC responses and SLE, including the exacerbation of SLE by estrogen receptors and suppression of the disease by the glucocorticoid receptor and others. These findings have important implications for patients with SLE and have contributed to improved outcomes over the last few decades.

Several biological processes regulated by NRs, such as circadian rhythm or metabolism, also influence GC responses. Exactly how these processes influence NR regulation during GC response within defined cell populations such as Tfh, Tfrs, or GC B cells has not yet been investigated in detail. It will be of interest to determine whether NRs also play critical roles within Tfrs. This is important in light of the increasing number of compounds targeting NRs, some of which may be beneficial in the treatment of autoimmune diseases.

Without more knowledge about the receptor pathways involved in SLE patients, including alterations in nongenomic signaling, corepressor, and activator recruitment, better and more specific therapies involving NRs for treatment of SLE and other autoimmune diseases may not be possible.

## Conflict of interest

The authors declare no conflict of interest.

## Author contributions

WJO and NHK wrote the manuscript. BJ prepared the table and assisted in writing the manuscript.
